# Bioresorbable Vascular Scaffolds—Dead End or Still a Rough Diamond?

**DOI:** 10.3390/jcm8122167

**Published:** 2019-12-07

**Authors:** Mateusz P. Jeżewski, Michał J. Kubisa, Ceren Eyileten, Salvatore De Rosa, Günter Christ, Maciej Lesiak, Ciro Indolfi, Aurel Toma, Jolanta M. Siller-Matula, Marek Postuła

**Affiliations:** 1 Department of Experimental and Clinical Pharmacology, Centre for Preclinical Research and Technology, Medical University of Warsaw, 02091 Warsaw, Poland; matjezewski@wp.pl (M.P.J.); kubisa.michal@gmail.com (M.J.K.); cereneyileten@gmail.com (C.E.); mpostula@wum.edu.pl (M.P.); 2 Department of Medical and Surgical Sciences, Division of Cardiology, “Magna Graecia” University, 88100 Catanzaro, Italy; saderosa@unicz.it (S.D.R.); indolfi@hotmail.com (C.I.); 3 Department of Cardiology, 5th Medical Department with Cardiology, Kaiser Franz Josef Hospital, 31100 Vienna, Austria; guenter.christ@wienkav.at; 4 1st Department of Cardiology, Poznan University of Medical Sciences, 1061701 Poznań, Poland; maciej.lesiak@skpp.edu.pl; 5 Department of Internal Medicine II, Division of Cardiology, Medical University of Vienna, 231090 Vienna, Austria; aurel.toma@meduniwien.ac.at

**Keywords:** bioresorbable vascular scaffold, drug-eluting stent, percutaneous coronary intervention, angioplasty, acute coronary syndrome

## Abstract

Percutaneous coronary interventions with stent-based restorations of vessel patency have become the gold standard in the treatment of acute coronary states. Bioresorbable vascular scaffolds (BVS) have been designed to combine the efficiency of drug-eluting stents (DES) at the time of implantation and the advantages of a lack of foreign body afterwards. Complete resolution of the scaffold was intended to enable the restoration of vasomotor function and reduce the risk of device thrombosis. While early reports demonstrated superiority of BVS over DES, larger-scale application and longer observation exposed major concerns about their use, including lower radial strength and higher risk of thrombosis resulting in higher rate of major adverse cardiac events. Further focus on procedural details and research on the second generation of BVS with novel properties did not allow to unequivocally challenge position of DES. Nevertheless, BVS still have a chance to present superiority in distinctive indications. This review presents an outlook on the available first and second generation BVS and a summary of results of clinical trials on their use. It discusses explanations for unfavorable outcomes, proposed enhancement techniques and a potential niche for the use of BVS.

## 1. Introduction

Cardiovascular disease is the most common cause of death, and by the year 2030, up to 44% of the adult US population is projected to suffer from some form of it, including ischaemic heart disease and acute coronary syndromes [1]. The effective restoration and maintenance of coronary vessel patency is a major problem requiring evaluation [2]. The idea of vascular restoration after the implantation of coronary stents has projected the development of bioresorbable vascular scaffolds (BVS) at the forefront of technological advancement in the field of coronary devices [3,4,5,6]. The design of BVS was prompted in an attempt to solve the limitations of durable drug eluting stents (DES), including (i) the occurrence of very late stent thrombosis (VLST), (ii) late expansive and adaptive vessel remodelling, (iii) anatomical limitations in case of surgical revascularization and (iv) impairment of computer tomography imaging [7,8]. After the initial excessive enthusiasm around BVS, the community was overly disappointed by the results of clinical trials. Complete resorption and improved vasomotor response of first generation of BVS which were believed to result in a reduced risk of target lesion failure (TLF) and stent thrombosis (ST) have been largely questioned [9,10,11,12,13]. In particular, the resorption time for the first commercially available BVS, ABSORB, has turned out to be substantially longer than initially thought. Therefore, it has been hypothesized that the resorption process itself or its delay can trigger complications [14,15,16]. A poor safety profile, especially in terms of target vessel myocardial infarction (TVMI) and an ST of 3 year follow up in ABSORB, as well as the negative results of the Amsterdam Investigator-Initiated ABSORB Strategy All-Comers Trial (AIDA) induced the manufacturer, Abbott Vascular, to halt the commercialization of ABSORB BVS [17,18,19]. Similarly, despite good outcomes of the BIOSOLVE I trial, the DREAMS BVS is still not ready for clinical use as the sparse data available stem from a small number of nonrandomized studies, conducted on a small number of patients [20,21,22,23]. Nevertheless, thanks to the encouraging results from the BIOSOLVE II and III studies which report very good outcomes in the 184 patients enrolled, with a more complex anatomical setting, we are facing a steady rise in the clinical use of the Magmaris stent within the ongoing prospective registry BIOSOLVE IV [24,25].

Overall, BVS appear to be a ‘critical’ development phase, and the currently clinically available BVS were given a class III indication for clinical use outside of studies in the current European Society of Cardiology (ESC) guidelines [26]. However, the disappointing outcomes mentioned above derive from studies in which optimal implantation strategies, proper imaging and long and potent platelet inhibition have not been extensively applied. On the contrary, it is proved that proper assessment of the target vessel segment with intravascular ultrasonography (IVUS) or optical coherence tomography (OCT) together with pre- and/or post-dilation can efficiently improve safety profile of BVS [27,28,29]. Thus, it is highly possible that refined second generation scaffolds with optimized implantation technique and proper imaging may restore the position of BVS and be competitive towards DES.

Our systematic review discusses the hypothetical advantages of BVS in the light of disappointing results obtained so far in clinical trials. Additionally, we aimed to explore possible methods to improve BVS performance starting from the design of next generation BVS and ending with procedural and pharmacological highlights.

## 2. Potential Advantages of BVS over Current Generation DES

**Figure 1:**
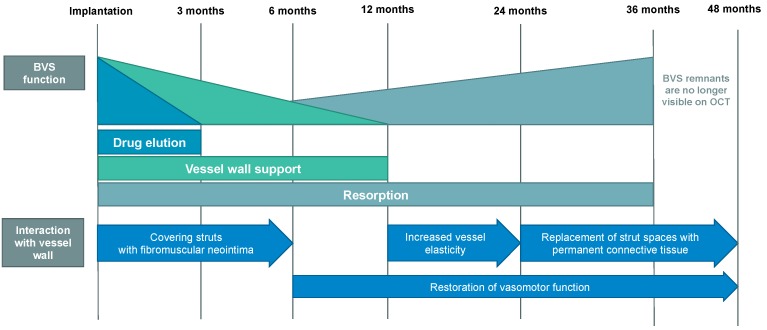
Timeline of bioresorbable vascular scaffolds (BVS) resorption and its interactions with vessel wall [6,16]. Presented course of phases and events is a generalization for available BVS. Certain time points are specific for ABSORB BVS-see Table 1. Abbreviations: BVS, bioresorbable vascular scaffold; OCT, optical coherence tomography.

Fully bioresorbable stents consist of synthetic biodegradable polymers that are intended to initially display functions similar to DES, and then dissolve within months after implantation, which may lead to the restoration of vasomotor function. In order to hold their promise, BVS should provide all potential advantages without sacrificing too much in terms of performance in comparison to DES. Another noticeable phenomenon (unrealistic using a solid metal stent) is the restoration of endothelial function with secondary reduction of atherosclerotic plaque [30,31]. After dissolving, it allows to maintain the integrity of the artery and return to its physiological properties (systolic and diastolic), thereby facilitating a beneficial remodeling and, consequently, causing a reduced passage for persistent inflammation (Figure 1) [31,32]. Therefore, a hypothesis has been put forward regarding the benefits of BVS, especially in younger patients or those with acute coronary syndromes, in which the metal stain is less likely to heal [33]. Among other features, the unfailing of the covered side branches after resorption, as well as avoiding the effect of a ‘full metal coat’, especially during diffuse disease, were also foreseen, providing early treatment of restenosis in the stent without additional layers of metal stents occupying the space [34]. BVS also give the possibility of a surgical revascularization procedure [31]. Moreover, this new technology also has significant benefits in the patient’s personal preferences to avoid having a permanent foreign body [32]. However, data available so far show us that most promises associated with the advantage of resorption had been overestimated. In fact, an ultimate demonstration of most potential advantages of BVS, such as restoration of the physiological function of vessels and endothelium, and the possibility of future surgical interventions within the same lesion, are still lacking [35]. 

## 3. Overview of First and Second Generation BVS

The first generation of BVS was initiated by ABSORB and DESolve scaffolds based on poly-L-lactic acid (PLLA) and DREAMS G1 scaffold based on magnesium [36,37]. Second generation of BVS embraces constantly expanding variety of scaffolds with enhanced properties and novel features. PLLA-based ART (Terumo, Tokyo, Japan) and DESolve Cx plus, the tyrosine analogue-based Fantom (REVA Medical, Inc., San Diego, CA, USA) and the magnesium-based Magmaris (Biotronik, Berlin, Germany) were introduced to clinical practice. The general characteristics of BVS are presented in Table 1. Yet, the variety of other devices based on polymers, metallic alloys or their combination remains in development [38,39]. Their development originates from an attempt to arrange a scaffold with thinner struts. The diameter of struts may not exceed 100 µm, whereas in the first generation it ranged from 150 µm [40]. The reduced thickness is believed to cause less blood flow disturbances and therefore to be associated with a lower risk of in-ST and a shorter requirement of dual antiplatelet therapy (DAPT). Additionally, tiny meshes become covered with a thinner layer of neointima and protected from the narrowing of the vessel lumen [41]. The newest devices are made of either magnesium alloy or polymers, including derivatives of PLLA and deaminotyrosine polycarbonate. These materials ensure greater resistance to fractures during post-implantation dilation and attain mechanical properties comparable to ordinary DES [30,42,43,44].

The ABSORB stent has a PLLA backbone, strut thickness is about 150 μm, with a bioresorbable coating of poly-D,L-lactic (PDLLA) with a thickness of 7 μm, secreting everolimus with a similar pharmacokinetics to the Xience DES [31,34,35,45,46,47,48,49]. Due to the presence of ester bonds between the PLLA and PDLLA monomers, degradation occurs by stepwise hydrolysis. In the final stage, either PLLA or PDLLA particles degrade entirely to lactic acid, or remnants smaller than 2 μm are phagocytized by macrophages [31,50]. Degradation is a mild, progressive process with minimal inflammatory reaction [51]. In order to obtain the appropriate mechanical framework of these stents, it became necessary to increase the thickness of the strut, because of lower tensile strength, reduced stiffness and the chance of deformation [35,52]. Studies have shown that in order to avoid strut rupture or abnormal decomposition, ABSORB stents require accurate lesion, judicious patient selection and an appropriate implantation technique, as they are able to stretch up to 0.7 mm beyond the nominal diameter [36,53]. ABSORB stents are radiolucent and therefore may not be visualized in fluoroscopy. For this reason, the stent at both ends was provided with two platinum markers, present to allow radiographic recognition. However, due to their tiny dimensions they identification requires high quality fluoroscopic imaging [52].

DESolve has a similar strut thickness (150 μm in the first generation), is composed of a PLLA-based scaffold and equipped with two platinum-iridium markers to enable radiographic visualization. The second generation, DESolve Cx plus, has a strut thickness of 120 µm, with a length of 14 to 28 mm and a diameter of 2.5 to 4.0 mm [54]. In the initial version, it eluted novelists at a rate of 80% during a month after implantation [55,56,57]. High flexibility enables extension up to 5 mm, without risk of breakage and provides greater radial strength in the vessel during the critical period of up to 4 months after implantation. In addition, DESolve scaffolds presents were observed of passively expand within 1 h after implantation, whereas ABSORB stents present a tendency to recoil [56,57]. However, under experimental conditions it was noticed that this ‘autocorrect’ feature is able to generate only small radial forces, so that it improves stent positioning, but does not exert a relevant impact on the vessel wall [58]. It has been hypothesized that it may either contribute to the reduced risk of malfunction of the stent immediately after implantation or may prove to be a beneficial feature in acute myocardial infarction, where the stent may be undersized. However, there is no data to support this concept [54]. The new model also promises biodegradation in the first year by as much as 95% with the assumption of full resorption of the stent up to 2 years [54,56,59]. After this period, the polymer is replaced with a loose net mainly composed of proteoglycan, followed by a new connective tissue [54]. DESolve scaffolds differ, therefore, from ABSORB stents due to the properties of self-expansion and increased tolerance to excessive stretching. 

DREAMS G1 stent is based on a frame made of absorbable magnesium alloy. Magnesium attains better mechanical properties such as a higher capacity of elongation and an increased tensile strength, allowing to use a thinner strut structure [54,60]. Due to the intrinsic radiolucency of magnesium and lack of markers mounted, the first generation stents are not visible in conventional imaging, but at the same time they are compatible with magnetic resonance imaging (MRI). These stents have bioresorbable coating of poly-lactic-co-glycolic acid (PLGA) eluting paclitaxel, and decompose in approximately 3–4 months [20,22,61,62,63]. The absorption of the magnesium alloy is a two-stage process, starting on the luminal surface of the scaffold, progressing towards the layers, until only the trace of hydroxyapatite remains at the site of implantation. In addition, magnesium reacts with water to form magnesium hydroxide, which starts the corrosion process [56]. Corrosion, however, does not proceed to the same extent on all sides, preferring the lateral surfaces of the struts. The initial crystal structure of magnesium hydroxide is gradually transformed into an amorphous body with a high-water content. After a time, the material is absorbed again through the core-infiltration of the cells [20]. In addition, some in vitro studies have shown that elevated magnesium concentrations in the coronary arteries per se reduce smooth muscle cell proliferation and increased endothelial cell proliferation [60,64]. Another advantage of these devices was the evidence of lower thrombogenicity in animal studies [60]. Despite the anti-arrhythmic properties of magnesium and its inhibitory effect on the release of endothelin-1, no adverse effects have been observed due to stent degradation [20,65]. The next generation, DREAMS 2 BVS (Magmaris), was enhanced in following ways: (i) its coating was thickened from 1 µm to 7 µm and converted from PLGA to PLLA, (ii) paclitaxel was substituted with sirolimus with greater elution (1.4 μg/mm^2^), and (iii) the strut thickness increased to 150 µm × 140 µm [61].

The main highlight of the Fantom scaffold is its intrinsic radio-opacity. The presence of iodinated tyrosine analogue polymer enables more precise deployment and non-invasive radiological assessment throughout the whole degradation time. The content of iodine in a single device is negligible in comparison with the amount administered in a contrast media [38].

## 4. Real-World BVS Performance—Outcomes and Evaluation

**Figure 2:**
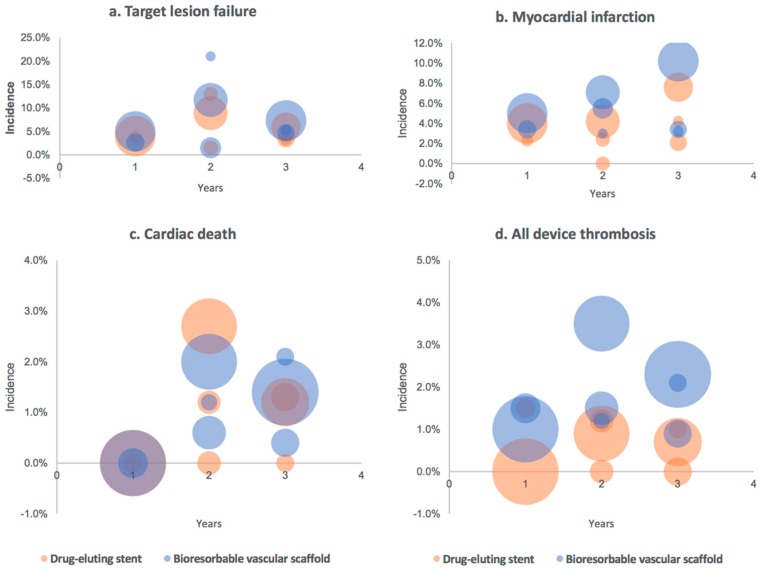
Comparison of incidence rate of major adverse events in randomized trials: (**a**) target lesion failure, (**b**) myocardial infarction, (**c**) cardiac death and (**d**) all device thrombosis. Figure presents data from ABSORB, AIDA, EVERBIO II and TROFI II trials. The circle diameter represents the number of patients in respective trials [17,19,66,67,68,69,70].

The safety and efficacy of BVS devices in clinical trials are presented in Figure 2 and Table 2. As for now, the superiority of any BVS over DES has not been shown in a randomized trial.

The first data describing BVS performance stems from a single arm ABSORB I study comprising of a 5 year observation of 130 patients in total. Safety and efficacy were assessed by observing events of ST and occurrence of major adverse cardiac effects (MACE). MACE was defined as nonfatal stroke, nonfatal myocardial infarction and cardiovascular death. The 5 year results were promising, inasmuch as in both cohorts no ST was observed, and the MACE rate ranged from 3.4% to 11% in cohorts A and B, respectively. It indicated that ABSORB BVS has a potential to overtake DES in the terms of safety profile and led to trials directly comparing ABSORB BVS with Xience V DES [31,72]. ABSORB II and ABSORB III studies were prospective, randomized, single-blind, multi-center trials which aimed on proving the non-inferiority of ABSORB BVS versus drug eluting Xience V DES. The primary endpoints of the study enclosed comparison of cardiac death (CD), TVMI and target lesion revascularization (TLR) rates one year after implantation, while secondary endpoints included the assessment of primary endpoint parameters at 2–5 years’ time and the assessment of ST, VLST and cost-related data.

The ABSORB II trial was the first to report inferiority of ABSORB BVS. 3 year follow-up was associated with a two-fold greater risk of TLF in comparison with Xience V (10% vs. 5%; *p* = 0.0425) [17,80]. Later, 2 year and 3 year observations in ABSORB III trials demonstrated ABSORB BVS inferiority in terms of overall ST and TLF driven mainly by TVMI [66,67]. Finally, the preliminary 30 day results of the most up to date and most populous ABSORB IV study revealed lower acute device success rate (94.6% vs. 99.0% (*P* < 0.0001)), greater risk of TLF (5.0% vs. 3.7%; *p* = 0.02) and greater ischemia-driven target vessel revascularization rate (ID-TVR) (1.2% vs. 0.2%; *p* = 0.003) [68]. Simultaneously, cumulative meta-analyses embracing ABSORB II, III, AIDA, EVERBIO II and TROFI II trials indicated the superiority of DES in the terms of both TLF and overall ST [9,10,69,70]. So far, only two country-specific trials (ABSORB JAPAN and ABSORB CHINA) demonstrated superiority of ABSORB BVS over Xience V DES [71,74]. The published meta-analyses and the results of the ABSORB IV trial formed the basis of the decision to cease production of ABSORB BVS in late 2017.

DREAMS G1 performance was assessed in 1 and 3 years’ long observation in the course of the BIOSOLVE I study [21,23]. The BIOSOLVE I trial included 46 patients with silent ischemia, stable or unstable angina and assessed angiographic and IVUS follow-up at 6 and 12 months together with 3 years’ clinical follow-up. In opposition to ABSORB trials, proper implantation strategy elements such as pre-dilatation were compulsory [20]. TLF occurrence at 6 months and 12 months is presented in Table 3. While no cardiac deaths or ST events were observed, the final results available in 3 year follow-up indicated 6.6% of TLF, 4.3% ischemia driven target lesion revascularization (ID-TLR) and 2.2% TVMI. Based on the angiographic results, it was assumed that DREAMS still could not compete with the 3rd generation of DES. BIOSOLVE-II and BIOSOLVE-III trials have been designed to observe its performance 3 years after implantation. As for now, data is available for 184 patients with single and multiple lesions in up to 2 years’ follow-up. Pooled analysis concluded with no trace of thrombosis, and TLF occurred in 3.3% and 5.9% of patients in the BIOSOLVE-II and BIOSOLVE-III groups respectively. It is worth noting that four cardiac deaths were observed among both groups [22,24].

BIOSOLVE-IV—a prospective, observational trial—is currently ongoing and aims at enrolling 2054 patients to be followed-up for 5 years. First year outcomes for the first 400 patients were recently published [25]. Procedural success was achieved in all but three patients. Target lesion failure (TLF) (primary endpoint) was registered in 4.3% of patients, and was exclusively composed of target lesion revascularizations. The rate of target vessel myocardial infarction was 0.88% and a single definite scaffold thrombosis was reported (0.3%) 10 days after implantation in a calcified lesion after a 5 day interruption of DAPT to perform a surgical minimally invasive revascularization of a non-target vessel.

Despite DESolve’s greater self-expansion properties and its unique self-correction property, which helps to avoid malapposition, no study proved its superiority over DES [54]. In addition, recent observational studies indicated propitious results [76,77]. Nevertheless, a non-complete follow-up represents the study’s limitations in long-term DESolve performance and the risk of late complications, e.g., VLST has not been properly investigated yet. As a consequence, the first generation of DESolve did not find application on the market. As such, the next generation, DESolve CX plus, has been halted.

The performance of Fantom scaffolds has been examined in two studies: Fantom I and Fantom II, including 7 and 117 patients respectively. The Fantom I pilot trial resulted in preserved patency in target vessels observed in IVUS after four months and no cardiac events after six months [78]. The multi-centre Fantom II trial observed short-term procedural success in 99.1% of cases, a 2.8% rate of MACE and one event of in-ST after six months. Investigators and commenters find these results comparable with other BVS. More encouraging results are anticipated from Cohort B of the Fantom II study, including 240 patients and nine month follow-up [79,82].

## 5. Anatomy of Failure: Explanation for Unfavorable Outcomes

**Figure 3:**
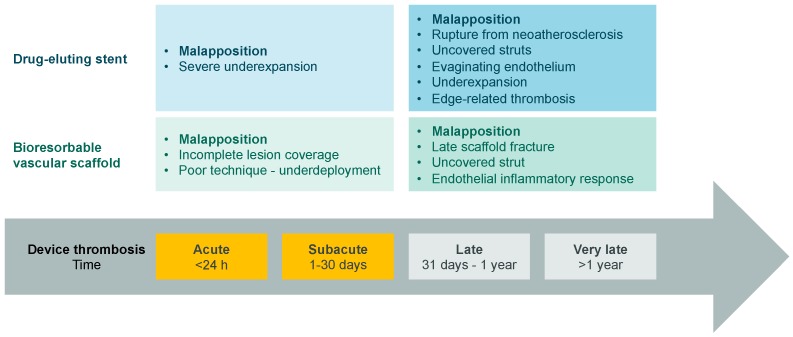
Comparison of the causes of device thrombosis in drug eluting stents (DES) and bioresorbable vascular scaffolds (BVS) [12].

As for now, the superiority of BVS over DES has not been shown in a randomized trial. A series of large-scale, post-registration studies showed long-term performance to be influenced by factors on every step of application of BVS, i.e., from device design to procedural specifics and vascular properties at the site of implantation (Figure 3) [83,84]. Optical analysis of intraluminal changes brings closer an explanation of the inferiority of BVS in clinical trials. Firstly, BVS implantation correlates with a greater asymmetry index (AI) and eccentricity index (EI) of the vessel in long term follow-up [85]. EI is defined as the ratio of minimum and maximum scaffold/stent diameter per cross section, while AI is defined as the ratio of minimal to maximal device diameter [85]. The greater the AI and EI value, the less symmetric the vessel section. Greater AI and EI were the consequence of the fact that ABSORB BVS is ‘less forgiving’ in case of inadequate deployment technique. Improper implantation, and consequently greater AI and EI, was determined to have a direct and significant impact on TLF occurrence in the ABSORB group [85,86]. Additionally, in numerous trials, the implantation of ABSORB correlated with lower minimal lumen diameter than Xience V, more extensive vessel remodeling, greater late luminal loss and decreased mean lumen area [17,87,88].

Furthermore, as shown by randomized studies, after the implantation of bioresorbable stents, a lower minimal diameter of the vessel lumen significantly increased initial stenosis which results in a greater risk of recoil in vivo [53,81,89]. The mentioned phenomena were caused by an increased strut thickness, greater than in DES; bulky, discontinuous, malapposed struts; structural disruption; and finally, incomplete resorption (even in 3 year observation time (the PRAGUE-19 trial)) [73,86]. Mentioned factors led to greater neointimal hyperplasia, greater volume of intraluminal masses and eventually greater coronary artery lumen narrowing, resulting in device failure [90,91]. Thus, it is highly probable that the failure of ABSORB was caused by a combination of faulty device design and a far from optimal implantation technique.

BVS are much more demanding in terms of the implantation technique and require specific protocol called the ‘PSP technique’ (Prepare the lesion, Size adequately, Post-dilate). Prior to implantation, it is necessary to perform an in-depth target segment imaging, in order to accurately assess the dimensions of the vessel and detect the presence of possible calcification [20,32,35,92,93]. Recent studies and reconsideration of the results of five ABSORB studies correlate with accurate adjustment of a scaffold and optimal post-dilation with a lower risk of TLF, and excessive pre-dilation with a lower risk of ST [94,95,96]. Therefore, BVS should not be implanted in places which cannot be accessed with a pre-dilating balloon. In next step, the scaffold should be expanded gradually by means of a five second pressure increase by two atmospheres, and the target pressure should be maintained for 30 s [32]. On the contrary, proper positioning of DES is a one-step procedure with limited time of ischemia [20]. However, prolonged ischemia appears not to impact BVS efficacy, and their use may be warranted even in chronic total coronary occlusions [97].

Recent studies have identified clinical scenarios which require particular attention. Desirable post-dilation steps cannot be performed safely on scaffolds longer than 28 mm, due to the risk of breakage. Therefore, long lesions demand a specific ‘scaffold to scaffold’ technique to avoid overlapping, resulting in impaired radial strength [98]. The use of BVS in lesions located on vessel bifurcations is associated with an acceptable risk of TLR, however, still greater than in use of DES [99]. Eventually, BVS are contraindicated in aorto-ostial lesions [100]. Acute coronary syndromes seemed to be a propitious target for BVS due to feature of restoration of the native vessel lumen and vasomotor function. This indication is supported by the results of randomized TROFI II and ISAR- ABSORB MI trials on STEMI patients treated with either ABSORB BVS or DES, which concluded with a comparable healing score and risk of adverse events [70,75].

The first-generation BVS ABSORB seems to increase the risk of ST and TVMI as shown by recent meta-analyses [101,102,103,104,105]. Furthermore, analysis of the data collected by several registries shows that this BVS associates with a high rate of early (within 30 days) definite or probable ST of 1.3–1.5% [106,107]. Moreover, 1 year after implantation, the definite or probable ST rates are markedly higher (up to 3.1%) than the rates detected by the randomized trials [108,109,110,111,112,113,114]. This apparently higher thrombogenicity may be caused by two major limitations of the first-generation thick-strut poly-lactic acid BVS. First, mechanical limitations of the BVS demand a more elaborate percutaneous coronary intervention (PCI) technique [115]. This may increase the risk of suboptimal scaffold implantation. Second, structural limitations of the BVS lead to the creation of laminar flow disruptions [116]. This may serve as a nidus for inter-strut thrombus formation.

**Figure 4:**
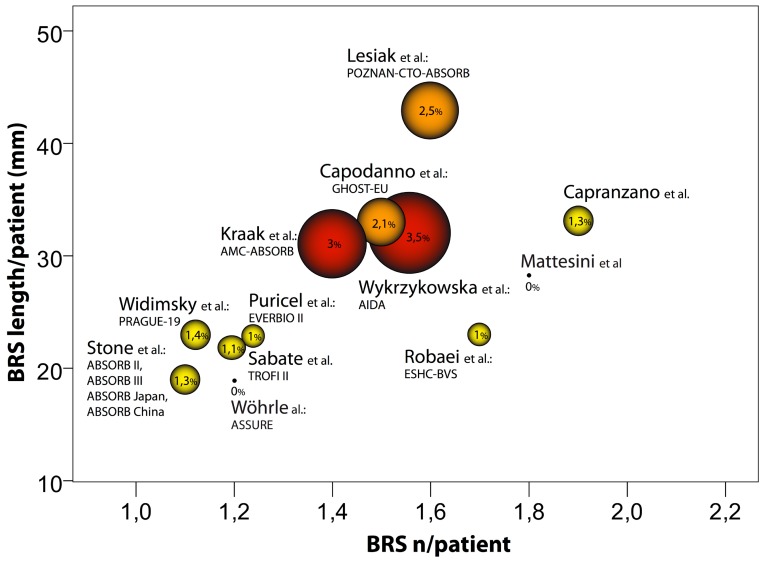
Bubble graph showing the relationship between the mean number and length of the implanted scaffolds and the incidence of scaffold thrombosis at the 6–12 month follow-up. Only randomized controlled trials, or registries with ≥100 patients that reported the required scaffold implantation details and had a follow-up duration of 6–12 months were included. The circle diameter indicates the reported percentage of definite or probable scaffold thrombosis [19,80,81,102,107,112,113,114,117,119,120,124].

The mean number and length of BVS implantation and its relation to the incidence of ST is illustrated in Figure 3, based on data from randomized controlled trials and registries with ≥100 patients that report a 6–12 month follow-up and BVS details [81,101,102,103,104,106,107,108,109,110,111,112,113,114,117,118,119,120,121,122,123]. Only the ASSURE registry, which included 183 patients, reported no cases of ST at 1 year follow-up [119]. However, the mean number and length of the implanted BVS in the ASSURE registry were comparable to those in the selected patient populations of the randomized controlled trials. Thus, it suggests that the cohort of the ASSURE registry was rather a low risk population and that this may account for the low event rate that has been observed [102]. Figure 4 illustrates that there is a trend toward an association between the stent length/number of implanted stents per patient and the incidence of ST. Since late ST is a crucial concern in the use of BVS, a long-term (at least 3–5 years) observation period should be warranted in upcoming trials.

## 6. Niche for BVS and Optimization of BVS Action

The excellent performance of DES makes them a tough benchmark to beat. The current generation of DES provides a very good vascular scaffolding with excellent radial strength. In fact, their use is associated with low rates of restenosis, ST or MACE [125,126,127,128]. Nevertheless, there are still concerns about their use [20,43,129]. The constant presence of a durable metallic prosthesis impairs vasomotor response vasodilation and causes a prolonged inflammatory reaction, which may eventually manifest in clinical observation [71,130,131,132,133]. Hence, BVS are believed to be distinctively beneficial in younger patients [73]. BVS may also serve patients’ personal preferences to avoid carrying a permanent foreign body [32]. BVS theoretically enable surgical revascularization at the site of implantation, however this concept has not been yet appraised in a clinical trial [31]. On the other hand, the group of preferred candidates is limited to those who clearly take advantage of the resolution of the scaffold. BVS are still discouraged in patients with limited life expectancy, inasmuch as a still uncertain safety profile prevails over the possible benefits associated with biodegradation [60].

The next generations of BVS resolving without antiproliferative drugs may overcome recent concerns about the use of DES in the management of peripheral artery disease. After years of satisfactory performance, the elution of paclitaxel is currently suspected to increase late mortality [134]. Despite the lack of an exact mechanism linking the action of an antiproliferative drug and all-cause mortality, the Vascular Leaders Forum convened by physicians, manufacturers and the Food and Drug Administration (FDA) in March, 2019 recommends cautious monitoring and reconsideration of alternative treatment options [135]. These circumstances may facilitate further development and show new indications for BVS.

The importance of routine pre-procedural imaging and strict compliance to the PSP technique should be highlighted [136,137]. The PSP technique is distinctively beneficial in the case of aggravated risk of thrombosis, e.g., in diabetic patients [124,138]. In line with this concept, the use of a longer inflation time, as suggested by the producer, allows better for device expansion and a more homogeneous apposition [53]. In order to select the proper size of BVS, the measurement of the target’s diameter was observed to be equally accurate under the guidance of either angiography or OCT [139]. Eventually, MRI enables non-invasive and non-radiative assessment of BVS patency after implantation and may be an alternative for patients with recurrent angina after angioplasty [140]. Despite the design of comparable radial strength of BVS to DES, it was noted that excessive stretching decreases its radial strength and elasticity and consequently leads to breakage [32,115,141]. This observation was confirmed in an experiment performed on a computational model. An arrangement of a mesh and the dimensions of a single strut determine the specific post-dilation size of a scaffold at which optimal mechanical and drug release properties are attained [142]. It is worth noting that available BVS devices are not equal in terms of their mechanical properties. PLLA-based scaffolds attain greater bending stiffness but lower radial strength, and tend more to recoil than magnesium-based scaffolds [58]. Among polymer-based scaffolds, the DESolve device attains greater flexibility and adapts better adaptation to the shape of the vessel [57,143]. These features are likely to contribute to upcoming studies as specific indications for certain devices. Greater radial strength may be utilizable in large vessels, while greater elasticity in tortuous vessels.

Another important issue is the residual high platelet reactivity (HPR) under DAPT, which represents one of the major determinants for worse outcomes of patients after PCI [144,145,146,147]. Therefore, the existence of a therapeutic window of platelet reactivity for P2Y_12_ pathway inhibition was proposed [148]. Indeed, it has been shown previously in an all-comer PCI population that if both the P2Y_12_ and cyclooxygenase-1 pathway inhibition of DAPT are personalized using Multiplate (a “point of care” platelet function assay which determines multiple electrode platelet aggregometry), HPR rates improve along with the clinical outcomes of patients [149,150,151]. Therefore, it would be interesting to know whether personalizing DAPT, by intensifying the treatment in the case of HPR to P2Y_12_ receptor blocker and/or aspirin, would have an impact on the rates of ST after BVS implantation. Personalization of DAPT remains a controversial issue because the randomized trials on this subject had considerable shortcomings [152,153]. Nevertheless, it has been already shown that strict peri- and post-interventional optimization of platelet reactivity improves patient outcomes at 30 days in an all-comers PCI population who underwent metallic stent implantation [149,150,151]. It might be that optimizing platelet reactivity may be of even greater importance in BVS implantation, primarily because the thicker struts of the first-generation scaffold disrupt laminar flow and thereby increase the risk of thrombus formation [116]. In our opinion, not even the best implantation technique is capable of completely counteracting this inherent limitation of the current BVS. This suggests that to improve patient outcomes after PCI with this BVS, it might be necessary to optimize the platelet reactivity. Several catheterization laboratories intensify platelet inhibition through the routine prescription of the newer potent P2Y_12_ receptor blockers for a longer duration (up to 3 years) to reduce the rates of ST after BVS implantation. A currently ongoing study on the administration of ticagrelor after the use of BVS in coronary vessels is believed to elucidate this matter [154,155].

## 7. Conclusions

After the initial excessive enthusiasm around BVS stemming from the dream of disappearing stents, clinical trials brought a great disillusionment. Existing DES devices still remain first choice in majority of cases and at the moment it is hard to believe that the current BVS could jeopardize their position. The next generations of BVS are anticipated to overcome the limitations which emerged in the initial clinical experiences. In particular, the procedural complexity and higher than expected restenosis and thrombosis represent major limitations. In fact, both technological advances and clinical expertise are about to shed new light on application of BVS devices.

## Figures and Tables

**Table 1:** Summary of efficacy and safety of the bioresorbable vascular scaffolds in clinical trials. Poly-L-lactic acid, PLLA; poly-D,L-lactic, PDLA.

Device	Material	Year of Receiving CE Marking	Drug Eluted	Strut Thickness (µm)	Minimal Resolution Time (months)
First generation					
ABSORB	PLLA	2012	everolimus	156	>36
DESolve	PLLA	2014	novolimus	150	<24
DESolve Cx plus	PLLA	2017	novolimus	120	<24
DREAMS 1G	Magnesium alloy	2015	paclitaxel	120	9–12
Second generation					
Magmaris (DREAMS 2G)	Magnesium alloy	2016	sirolimus	120	9–12
Fantom	Tyrosine polycarbonate	2017	sirolimus	125	36
ART	PDLLA	2015	none	170	6

**Table 2:** Summary of the efficacy and safety of the bioresorbable vascular scaffolds in clinical trials. Target lesion failure, TLF; Target lesion revascularization TLR.

Study/Publication Date	Study Type	Follow-Up Time	No of Patients	No of Devices per Patient	Length of Devices (mm)	TLF (%)	ScT Definite/Probable (%)	MI (%)	TLR (%)	Cardiac Death (%)	Commercial Funding
ABSORB (Abbott, Lake County, IL, USA)											
ABSORB Cohort A [31]/Mar 2008	Observational	5 years	30	1	12 or 18	3.4	0/0	3.4	10.3	0	Abbott Vascular
ABSORB Japan [71]/Dec 2015	Randomized	1 year	400	1–2	8, 12 or 18	4.2	1.5/1.5	3.4	2.6	0	Abbott Vascular
ABSORB Cohort B [72]/Feb 2016	Observational	5 years	101	1	18	14.0	0/0	3.0	11.0	0	Abbott Vascular
PRAGUE-19 [73]/May 2016	Observational	3 years	113	1	<24	11.5	1.8/0.9	1.8	3.5	1.8	Abbott Vascular
ABSORB II [17]/Nov 2016	Randomized	3 years	335	1–2	<48	10	3.0/3.0	8.0	7.0	1.0	Abbott Vascular
ABSORB China [74]/Oct 2017	Randomized	3 years	238	1–2	<24	6.8	0.4/0.4	3.4	4.7	0.4	Abbott Vascular
ABSORB III [67]/Oct 2017	Randomized	2 years	1322	1–2	<24	3.7	1.9	1.3	2.6	0.5	Abbott Vascular
ABSORB III [66]/Dec 2017	Randomized	3 years	1322	1–2	<24	13.4	2.3	4.2	7.3	0.9	Abbott Vascular
ABSORB IV [68]/Oct 2018	Randomized	1 year	1296	1–3	>24	5	1	5	2	0	Abbott Vascular
AIDA [19]/Jun 2017	Randomized	2 years	924	1–2	N/A	11.7	3.1/0.4	7.1	7	2	Abbott Vascular
EVERBIO II [69]/Sep 2017	Randomized	2 years	78	N/A	N/A	21	1.2	3	23	1.2	Abbott Vascular, Biosensors International, Boston Scientific
TROFI II [70]/Nov 2018	Randomized	3 years	95	1	8, 12, 18 or 28	5.3	2.1	3.2	4.2	2.1	Abbott Vascular, Terumo
ISAR- ABSORB MI [75]/Dec 2018	Randomized	1 year	173	1	1	7.0	1.2/0.6	0.6	4.8	2.3	Abbott Vascular
DESolve NX (Elixir Medical Corporation, Milpitas, CA, USA)											
DESolve First-in-Man trial [59]/Jan 2014	Observational	1 year	15	1–2	14 or 18	6.7	0.8	6.7	6.7	6.7	Elixir Medical
DESolve 2 years [56]/Mar 2016	Observational	2 years	122	1	14 or 18	7.4	0.8	1.6	4.0	3.2	Elixir Medical
DESolve Cx [76]/Oct 2017	Observational	6 months	50	1	14, 18, 13 or 28	0	0	0	0	0	Elixir Medical
DESolve PCMF Study [77]/Nov 2018	Observational	12 months	102	1–2	14, 18 or 28	2,0	1.0/0	1,0	1,0	0	Elixir Medical
**DREAMS** (Biotronik, Berlin, Germany)											
BIOSOLVE-I [23]/Jun 2016	Observational	3 years	46	1	16	6.6	0	2.2	4.3	0	Biotronik AG
BIOSOLVE-II [22,24]/Sep 2016	Observational	2 years	118	1–2	≤21	5.9	0	0.9	3.4	1.7	Biotronik AG
BIOSOLVE-II and BIOSOLVE-III [24]/Jul 2017	Observational	6 months	184	1–2	≤21	3.3	0	0.6	1.7	1.1	Biotronik AG
**Fantom** (REVA Medical Inc., San Diego, CA, USA)											
Fantom I [78]/Apr 2016	Observational	4 months	7	1	18	0					N/A
Fantom II [79]/Sep 2017	Observational	6 months	117	1	18 or 24	2.6	0.9	1.7	1.7	0	REVA Medical

**Table 3:** Summary of incidence of primary endpoints in randomized studies comparing BVS and drug-eluting stents (DES).

Study	Compared Devices (No of Patients in Groups)	TVF RR/HR (95% CI)	Ischemia Driven TLR RR/HR (95% CI)	Cardiac Death RR/HR (95% CI)	TVMI RR/HR (95% CI)	Device Thrombosis Probable/Definitive RR/HR (95% CI)
ABSORB Japan [71]	ABSORB BVS vs. Xience DES (266/134)	1.15 [0.48, 2.72] *p* = 0.75	1.17 [0.31, 4.46] *p* = 1.00	N/A	1.51 [0.41, 5.47] *p* = 0.76	1.02 [0.19, 5.47] *p* = 1.00
ABSORB II [17]	ABSORB BVS vs. Xience DES (335/166)	2.11 [1.00, 4.44] *p* = 0.0425	1.65 [0.46, 5.92] *p* = 0.56	0.50 [0.10, 2.43] *p* = 0.56	5.70 [1.36, 23.87] *p* = 0.0061	N/A *p* = 0.0331
ABSORB China [74]	ABSORB BVS vs. Xience DES (236/235)	1.00 [0.51, 1.94] *p* = 0.99	1.66 [0.61, 4.49] *p* = 0.31	0.33 [0.03, 3.17] *p* = 0.37	2.99 [0.61, 14.65] *p* = 0.28	N/A *P* = 0.50
ABSORB III [66]	ABSORB BVS vs. Xience DES (1322/686)	1.41 [1.10, 1.81] *p* = 0.006	1.23 [0.85, 1.79] *p* = 0.27	1.17 [0.51, 2.69] *p* = 0.71	1.47 [1.02, 2.11] *p* = 0.03	3.12 [1.21, 8.05] *p* = 0.01
ABSORB IV [68]	ABSORB BVS vs. Xience DES (1296/1308)	1.35 [0.93, 1.97] *p* = 0.11	2.28 [0.99, 5.25] *p* = 0.0457	N/A	1.23 [0.84, 1.81] *p* = 0.29	4.05 [0.86, 19.06] *p* = 0.06
AIDA [19]	ABSORB BVS vs. Xience DES (924/921)	1.12 [0.85, 1.48] *p* = 0.43	1.17 [0.86, 1.58] *p* = 0.31	0.78 [0.42, 1.44] *p* = 0.43	1.60 [1.01, 2.53] *p* = 0.04	3.87 [1.78, 8.42] *P* < 0.001
EVERBIO II [81]	ABSORB BVS vs. Promus Element and Biomatrix Flex DES (78/160)	*p* = 0.12	*p* = 0.23	*p* = 0.55	*p* = 0.11	N/A
TROFI II [70]	ABSORB BVS vs. Xience DES (95/96)	*p* = 0.465	*p* = 0.678	N/A	*p* = 0.327	*p* = 0.55
ISAR-Absorb II [75]	ABSORB BVS vs. EES (173/89)	1.04 [0.39, 2.78]	0.84 [0.27, 2.57]	1.02 [0.19, 5.58]	0.51 [0.03, 8.20]	0.51 [0.07, 3.62]
